# Comparison of volumetric analysis between conventional and rotary files in the preparation of root canals in primary molars—an *in vitro* study

**DOI:** 10.3389/fdmed.2024.1489074

**Published:** 2024-12-24

**Authors:** Satish Vishwanathaiah

**Affiliations:** Division of Pediatric Dentistry, Department of Preventive Dental Sciences, College of Dentistry, Jazan University, Jazan, Saudi Arabia

**Keywords:** Kedo-S square, pediatric rotary files, Pro AF baby gold, primary molar, volume

## Abstract

**Background:**

To compare and evaluate the efficacy of canal preparation and volumetric filling of primary molars instrumented by conventional hand K-file, ProAF baby rotary file and Kedo-S square file using cone beam computed tomography.

**Materials and methods:**

Thirty freshly extracted human primary second molars were randomly divided into 3 groups of 10 teeth each. After access opening and working length determination, preoperative volume analysis was done using cone beam computed tomography (CBCT). The canals were then instrumented by either hand K-files, Pro AF Baby rotary files and Kedo-S square rotary files. Post operative volume analysis was performed using CBCT. All the canals were obturated using Metapex and scanned again using CBCT. Mean values of the pre- and post-operative canal volumes were analyzed using one-way ANOVA. Inter- and intra- group volumetric changes were analyzed statistically by *post hoc* test.

**Results:**

The mean difference in volume after canal preparation and obturation was the highest in the hand K-file group, followed by Pro AF Baby Gold group and the least in the Kedo-S square group. Inter and intra group comparison showed statistically significant differences for all the file groups used.

**Conclusion:**

Kedo- S square showed the least difference in preparation volume and better obturating volume compared to Pro AF baby gold file systems.

## 1 Introduction

The success of pulpectomy depends on both mechanical and chemical disinfection of the canal space of primary molars. Barr et al.'s endodontic revolution in pediatric dental practice had made a paradigm shift in performing pulpectomy in primary teeth (1, 2). Apart from iatrogenic errors, rotary NiTi files in pediatric endodontic practice had reduced the time spent on canal preparation with better cleansing of infected or necrosed pulp contents and efficient canal preparation which are curved and tortuous. Such canal systems make pulpectomy simple, quick, and cost effective without tiring the dental team, thereby restoring the tooth's integrity and function (3, 4).

Barr's contributions to endodontic files revolutionized root canal preparation by advancing rotary endodontics with nickel-titanium (NiTi) files. These files, known for the flexibility and adaptability to curved canals, enable efficient shaping while minimizing risks such as transportation, perforation, and excessive dentin removal. The variably varied tapered designs further improved canal preparation, reducing treatment time, and enhancing precision. Additionally, Barr's focus on minimally invasive endodontics (ME) emphasized preserving pericervical dentin, ensuring the long-term integrity of treated teeth, His innovations in file design aligned with contemporary approaches prioritizing conservation and efficiency, setting new standards for safety and clinical predictability in root canal therapy (5).

Effective and efficient preparation of canal space involves complete elimination of bacterial contents, with minimal removal of contaminated dentin and preserving the remaining dentin (6). This can be achieved by preserving the canal anatomy using file systems that maintain canal centering ability. Rotary file systems are designed specifically for these purposes which vary by length, cross sections, and flexibility (7).

ProAF baby comprises five file sequences and is made of heat-treated nickel titanium-controlled memory wire. They maintain a constant taper of 4% and 6% depending on the file sequence used. These files were designed to be similar to the file sequence used for permanent teeth (8). Kedo-S square consists of a single file system and is made of heat-treated Ni-Ti M-wire with titanium oxide coating. They have variably variable taper with a working length of 16 mm (9). The variably variable taper design of the Kedo-S Square file system refers to the taper variation within the file. This design addresses the unique anatomical challenges of primary teeth, including curved and tapering root canals, ensuring precise and efficient canal shaping. The taper changes along its length with varied taper between 4%-8%. The coronal segment has a larger taper to enhance debris removal and irrigation, the middle segment features a moderate taper for controlled shaping, and the apical segment employs a smaller taper to preserve dentin and reduce the risk of perforation or structural compromise. This design ensures better adaptation to canal anatomy, minimizes dentin removal, and reduces the risk of procedural errors such as canal transportation or file separation. Additionally, the enhanced taper in the coronal region facilitates debris evacuation and irrigation flow, improving the overall efficiency and safety of the procedure (9).

The study's rationale centers around these recently implemented file systems, which evolved to obtain optimal conditions for a pulpectomised tooth. This *in vitro* study aimed to compare and assess the efficacy of canal preparation and volumetric filling of primary molars instrumented with traditional hand K-files, ProAF baby rotary files, and Kedo S square files utilizing cone beam computed tomography. The null hypothesis postulated that rotary file systems were less effective than hand file systems in preparing the canals of primary molars.

## 2 Materials and methods

The study design was analysed and obtained from the institutional scientific review board of the College of Dentistry, Jazan University, Jazan. (CODJU-1706I). Forty freshly extracted human primary second molars were obtained from the out-patient pediatric dentistry clinic. A signed written informed consent was obtained from the parents or guardians regarding the use of the extracted tooth for research purposes which was also approved by the institutional ethical committee. The teeth were extracted due to the following reasons: non restorable crown structure, severe extraoral swelling and when parents were not willing to preserve the tooth by performing pulp therapies. According to the study's inclusion criteria, teeth had to have no or minimum physiological root resorption, which is specifically described as up to one-third of the root length from the apex. Teeth with calcified canals or those with root resorption greater than two-thirds of the root length were removed. The selection strategy was carefully designed to minimize confounding factors that might influence the study's findings, particularly those linked to notable root resorption or anatomical abnormalities.

The clinical significance of root resorption thresholds is particularly essential in endodontic therapy. Teeth exhibiting resorption exceeding one-third of the root length may possess inadequate structural integrity to facilitate effective root canal therapy, jeopardizing the procedure's long-term success. Excessive resorption might hinder the formation of an adequate seal in the root canal system, thereby elevating the risk of treatment failure or premature tooth loss.

### 2.1 Sample size estimation

$$\mathrm{Sample}\;\mathrm{Size}\;(n)=\frac{2S_{p}^{2}\;{\left[Z_{1-\alpha /2}+Z_{1-\beta }\right]}^{2}}{\mu_{d}^{2}}$$

$$S_{p}^{2}=\frac{S_{1}^{2}+S_{2}^{2}}{2}$$

where,

*Z* (1-*α*/2) = 1.64 for 90% confidence interval;

*Z*_1−*β*_ = 0.84 for 80% power;

*S*_1_ = 2.26 (standard deviation in Hand file group);

*S*_2_= 0.57 (standard deviation in Rotary file group);

µ*_d_* = 1.57(difference in mean ratio scores between two groups).

By substituting these values, the sample size was estimated to be fifteen (10) in each group. The study sample size was derived from an *in vitro* study by Singh et al. (9) with 95% power using G Power analysis. The total sample size was determined to be 45 teeth.

### 2.2 Preparation of the teeth samples

After the cleansing process using ultrasonic scalers, the teeth were stored in 0.5% sodium hypochlorite solution until its use in the *in vitro* study. For standardization of the samples for canal preparation, only mesiobuccal root of the primary second molars were considered for the study. The mesiobuccal root was used because it was one of the intact roots with minimal resorption. A total of forty-five teeth were collected, numbered and randomly divided into 3 groups of 15 teeth each. Access opening of the primary second molars were performed using a small round carbide bur in a high-speed handpiece. Any remnant necrotic coronal pulp was amputated using a spoon excavator. A size #10 K-file was used to determine the patency of the mesiobuccal canal. After confirming the patency of the canals, the canals were irrigated using 0.9% normal saline through a 31-gauge irrigation needle. The working length was established by subtracting 1 mm from visible length seen at the root apex.

### 2.3 Mounting of samples and pre-operative volumetric analysis

**Figure 1:**
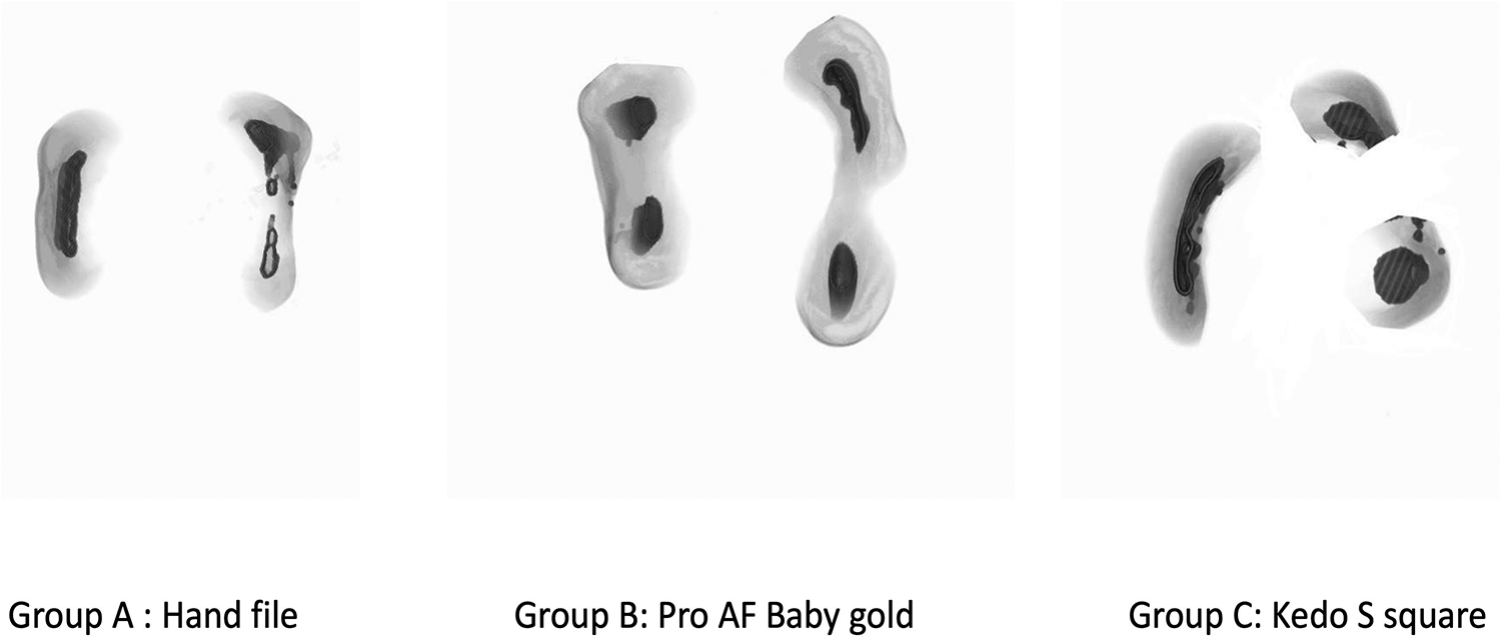
Pre-operative volumetric analysis.

**Figure 2:**
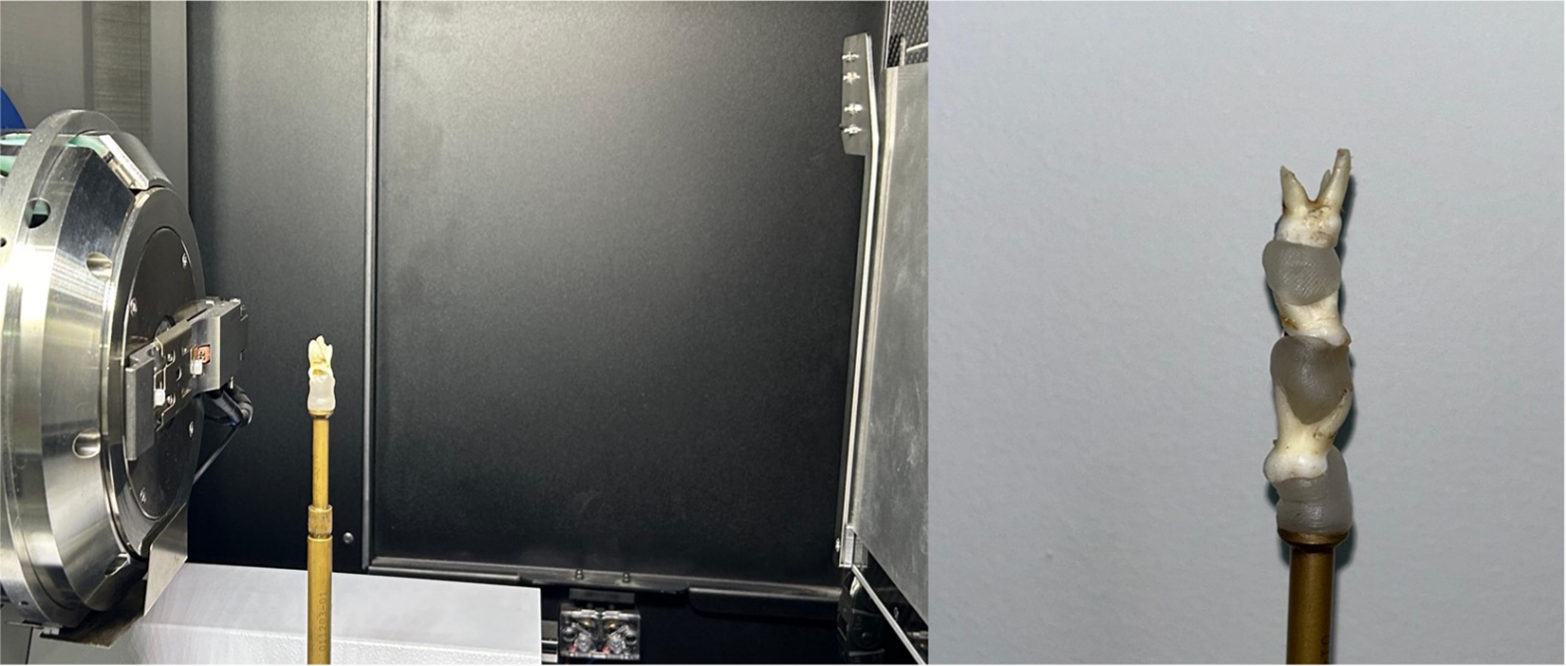
Mounting of the samples.

Preoperative volumetric analysis was done using cone beam computed tomography (CBCT) (Figure 1). All the samples were mounted in a vinyl polysiloxane impression material *(3M ESPE, GERMANY)* to form a template that was prepared for reciprocating the position both in the pre- and post-operative volumetric analysis. To maintain uniformity of the samples, the samples were arranged to make sure that the mesial surface of the teeth was on the right side as similar to the methodology performed from the previous study (11, 12). The samples were then subjected to light speed plus SCT scanner *(GE electricals. Wilwaukee, USA)* in an axial, coronal, and sagittal plane by an experienced operator who was blinded of the instrumentation sequence (Figure 2). Volume rendering and multiple planar volume reconstruction for root canal measurement were done using Advantage Windows Workstation Version V (GE System, Milwaukee, WI, USA). A constant thickness of 0.65 mm per slice and a constant spiral or table speed of 0.75 and 120 KVP was used. The volume of all the samples was calculated from the canal orifice to 1 mm short of apical foramen.

### 2.4 Root canal instrumentation

The instrumentation of the selected teeth was conducted by a single experienced investigator who routinely treats pediatric patients and hold expertise in utilizing manual and rotary instrumentation techniques.

Group A: The teeth samples were prepared using hand K-files (Mani, Tochigi, Japan). These hand K-files have an ISO standardized 2% constant taper with a working length of 21 mm. The canals were prepared till the determined working length of each sample using no. 15, 20, 25 and 30 size hand K-files in consecutive sequences. The files were regularly wiped using wet gauze to remove tissue debris. With every increase in file size, the canals were irrigated using 0.9% normal saline and 1% sodium hypochlorite to flush out the dentinal debris. Canal recapitulation was performed after the use of each file.
Group B: The teeth samples were prepared using Pro AF Baby Gold rotary files at 300 rpm at 2 N/Cm torque. Number 15 size hand K-file (Mani, Tochigi, Japan) was used to check patency upto working length. Bo file was used to instrument the first 3 mm beyond the orifice. B1 and B2 files were then used to complete the canal instrumentation up to the determined working length of the samples. Irrigation was performed using 0.9% normal saline followed by 1% sodium hypochlorite to flush out the dentinal debris.
Group C: The teeth samples were prepared using Kedo-S square rotary files (Kedo Dental, India) that has a variably variable (VV) taper at 250 rpm at 2.2 N/Cm torque. Number 15 size hand K-file (Mani, Tochigi, Japan) was used to check patency upto working length. Kedo-S square P1 (0.30/VV taper) file was used for instrumenting the canals up to the determined working length of the samples. Irrigation was performed using 0.9% normal saline followed by 1% sodium hypochlorite to flush out the dentinal debris.

### 2.5 post-operative volumetric analysis

**Figure 3:**
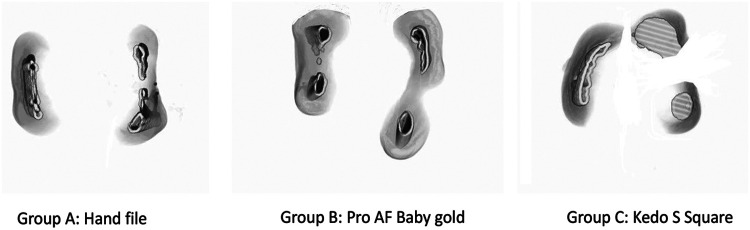
Post volumetric analysis.

All the canals were then dried using paper points before subjecting to CBCT (Figure 3). The samples in all the groups were again placed in the same template in the same position and scanned similar to the pre-operative volumetric analysis. Pre-operative volumetric analysis, the canal volume for each sample was measured from the canal orifice to 1 mm short of apex.

### 2.6 Obturation and post-obturation volumetric analysis

All the canals were obturated using Metapex (MetaBioMed) and entrance filling was provided using glass ionomer cement. The samples were placed back in the template and the final scanning was done. CBCT evaluation of all the samples in the pre-operative, post-operative and post-obturation analyses was done by an experienced radiograph analyst with 8 years of expertise in the field, who was blinded from the methodology that was used in the present study.

### 2.7 Statistical analysis

Mean values of the pre- and post-operative canal volumes after canal preparation, post-operative canal volumes after obturation and the differences in canal volume of all the samples were analyzed using one-way ANOVA. Inter- and intra- group volumetric changes were analyzed statistically by Tukey's *post hoc* test. A *p*-value of less than 0.05 was considered statistically significant. All the statistical analysis was done using Statistical Package for Social Studies (SPSS) v.22 produced by IBM, Illinois, Chicago, USA.

## 3 Results

The mean difference in volume after canal preparation was the highest in the hand K-file group, followed by Pro AF Baby Gold group and the least in the Kedo-S square group. Comparison within the groups showed statistically significant differences for all the file groups used (Hand K—*p* = 0.022; Pro AF Baby Gold—*p* = 0.034; Kedo-S square—*p* = 0.001) (Table 1). Inter-group comparison showed statistically significant differences between hand-K group and ProAF Baby Gold group (*p* = 0.025), ProAF Baby Gold group and Kedo-S square group (*p* = 0.011) & highly statistically significant differences between hand-K group and Kedo-S square group (*p* = 0.000) (Table 2). The mean difference in volume after obturation was the highest in the hand K-file group, followed by Pro AF Baby Gold group and the least in the Kedo-S square group. Comparison within the groups showed statistically significant differences for all the file groups used (Hand K—*p* = 0.017; Pro AF Baby Gold—*p* = 0.025; Kedo-S square—*p* = 0.000) (Table 3). Inter-group comparison showed highly statistically significant differences between hand-K group and Kedo-S square group (*p* = 0.000) & statistically significant differences between hand-K group and ProAF Baby Gold group (*p* = 0.046) and ProAF Baby Gold group and Kedo-S square group (*p* = 0.023) (Table 4).

**Table 1:** Mean pre- and post-operative volumes before obturation and difference in volumes of canals prepared under each group of files used in the present study.

Files Group	Pre-Operative Volume Mean ± SD (cm^3^)	Post-Operative Volume (before obturation) Mean ± SD (cm^3^)	Volume Difference Mean ± SD (cm^3^)	*p*-value
Hand-K	0.0051 ± 0.00042	0.0082 ± 0.00026	0.0031 ± 0.00012	0.022*
ProAF Baby Gold	0.0056 ± 0.00028	0.0078 ± 0.00047	0.0022 ± 0.00034	0.034*
Kedo-S square	0.0053 ± 0.00017	0.0071 ± 0.00015	0.0018 ± 0.00083	0.001*

* Statistically significant differences by Tukey's *post hoc* test.

**Table 2:** Intergroup comparison of mean difference in volumes of canals prepared before obturation.

Comparison between groups (before obturation)	*p*-value
Hand-K vs. ProAF Baby Gold	0.025*
Hand-K vs. Kedo-S square	0.000*
ProAF Baby Gold vs. Kedo-S square	0.011*

* Statistically significant differences by paired *t*-test.

**Table 3:** Mean pre- and post-operative volumes after obturation and difference in volumes of canals prepared under each group of files used in the present study.

Files Group	Pre-Operative Volume Mean ± SD (cm^3^)	Post-Operative Volume (after obturation) Mean ± SD (cm^3^)	Volume Difference Mean ± SD (cm^3^)	*p*-value
Hand-K	0.0052 ± 0.00034	0.0084 ± 0.00023	0.0032 ± 0.00016	0.017*
ProAF Baby Gold	0.0055 ± 0.00017	0.0080 ± 0.00046	0.0025 ± 0.00026	0.025*
Kedo-S square	0.0051 ± 0.00084	0.0074 ± 0.00074	0.0023 ± 0.00065	0.000*

* Statistically significant differences by Tukey's *post hoc* test.

**Table 4:** Intergroup comparison of mean difference in volumes of canals prepared after obturation.

Comparison between groups (after obturation)	*p*-value
Hand-K vs. ProAF Baby Gold	0.046*
Hand-K vs. Kedo-S square	0.000*
ProAF Baby Gold vs. Kedo-S square	0.023*

* Statistically significant differences by paired *t*-test.

## 4 Discussion

The results of the present study suggest that rotary files had more preparatory canal volume when compared to the hand file system. Kedo-SG Blue file system had better canal preparation volume than ProAF Baby Gold file system. Post-obturation analysis also suggested a superior obturating volume in rotary file systems, especially with Kedo-SG Blue, when compared to the conventional hand file system.

The results showed that primary root canal space had minimal preparation using both the rotary files compared to conventional hand file systems. This is because of the tip diameter, the Pro AF baby system used upto B2 file which has a 0.25 daumeter; Kedo S square cross section in the apical portion is thinner with a tip diameter of 0.28 whereas hand file's tip diameter of size 30 is 0.30. This result was similar to other studies who have reported that minimal preparation of canals was noticed using rotary file systems (13–15). Kedo-S square had the least mean difference in the canal preparation volume compared to Pro AF Baby Gold file system. This result was supported by the study done by Mohamed et al., who concluded that Kedo-S square had minimal dentin removal and preparatory volume (15). This was because Kedo S-square had two cross sections with minimal diameter and a variably variable taper design. This tended to minimally prepare the canals (16). This result was contradicted by study done by Swaminathan et al, who concluded that more dentin removal using Kedo-S file system as compared to MTwo file system (17). The taper design of the file can influence the preparation of the canal (18). Kedo-S rotary file systems using a VV taper (4%–8%) aided in improved coronal enlargement thereby facilitating a straight-line access and better flow of obturating material (3). More specifically, the utilization of a 0.25 tip with a 4% taper file proves essential for achieving adequate canal preparation in the apical and middle thirds. Simultaneously, the use of a 6% taper file in the coronal one-third enhances the overall preparation of the canal (12, 18). This could be the reason for better preparation in the Kedo-SG blue group. Post-obturation analysis showed that Kedo-S square had minimal obturation volume when compared to the other file systems used. This result was similar to the studies (19–21) who concluded that optimal obturation volume was obtained using Kedo-S square. This was due to the similar reason of two different cross sections and more taper in the coronal aspect that effectively provided a slight coronal flare to enhance the flow of the obturating paste through orifice (22).

Radiographic evaluation of canal preparation using hand or rotary instruments is essential to provide information on the efficiency of the instruments used. Such a non-invasive, cost-effective technique is by using CBCT which takes multiple two-dimensional images at different angulations to reconstruct a three-dimensional visual representation (23). This would be essential for a comprehensive assessment of the canal preparation for the operator to assess at ease. Earlier studies have proven that CBCT is an essential tool for measurements of before and after root canal preparation (24, 25). A recent systematic review on comparing CBT and micro-CT showed that there were no significant differences between both and CBCT can be as accurate as micro-CT in terms of assessment canal morphology (26).

No clear guidelines or design have been provided by any professionals for its use in primary teeth (1). Rotary file systems for permanent teeth have been redesigned for use in primary teeth. Pro AF Baby Gold file systems followed the regular 4% and 6% ISO standard taper that were commonly used for canal preparation in permanent teeth. The only difference was that the length of the file was 16 mm and the active cutting length was around 12 mm. Kedo-S brand was the first rotary file system designed specifically for its use in preparing canals of primary teeth. With variably variable taper design and cutting length of the file designed specifically to the length of primary teeth, the Kedo-S file system tends to be an ideal rotary system for mechanical disinfection of root canals (27). Kedo-S square have two cross sections combined into a single file which are both heat treated, and titanium oxide coated (20). Such patented designs would require assessment for efficient canal preparation for its clinical usage. Thus, a CBCT analysis was performed to evaluate the efficacy of both the rotary file systems. Obturating the prepared canal space will prevent the re-entry of contaminants and provide a 3-dimensional fluid tight seal thus providing long term success of the endodontic treatment (28, 29). Hence the assessment of the obturation volume was one of the objectives of the present study.

Chemo mechanical preparation is the most practiced achieving complete disinfection of canal space with elimination of microorganisms and thereby preventing the infection to peri radicular tissues. This protocol involves both mechanical filing and intermittent use of mitigating solutions to successfully perform pulpectomy that favours periapical healing (30). Although irrigants play a vital role, this study is more inclined towards the mechanical systems used for cleansing the canal space. Conventional hand instruments that have been used for decades for biomechanical preparation of root canals have had its own drawbacks. Ribbon shaped, curved and tortuous canals of primary teeth are left underprepared due to stiffness of the hand file systems that compromises the complete disinfection of the canal anatomy (31). Ledge formations and canal transportations along the coronal concavity and along the radicular convexity are commonly noticed when curved canals are intervened using stiff hand files (20) With the protocol put forward by Barr et al., and Priyadarshini et al., these complications were avoided by using rotary file systems in primary teeth (31, 32). The current study was thus designed to inspect the effectiveness and volumetric changes using hand files and two pediatric rotary file systems designed for primary teeth suggest that Kedo-S square could help in preservation of dentin with minimal canal preparation thereby avoiding thinner dentin regions commonly seen at the furcation.

Utilizing a conservative preparation volume in primary teeth is essential for safeguarding dental structure, upholding tooth integrity, and securing best long-term results for the developing dentition. Deciduous teeth, characterized by weaker enamel and dentin, are susceptible to fractures if an excessive amount of tooth structure is extracted. Moreover, their enlarged pulp chambers render them vulnerable to pulpal exposure, perhaps leading to inflammation, infection, or requiring more invasive procedures such as pulpectomy. A conservative approach preserves the functional and structural integrity of these teeth, which are crucial for mastication, speech development, and facilitating the appropriate eruption of permanent teeth. Conservative preparations also reduce microleakage by maintaining natural anatomical shapes, enhancing the fit of restorations, and diminishing the risk of secondary caries. Maintaining crown structure is essential for arch stability and provides sufficient room for the eruption of permanent teeth, hence preventing malocclusion. Moreover, minimally invasive procedures improve the child's comfort and participation, hence diminishing oral anxiety and the necessity for sophisticated behavior control strategies. Conservative preparations enhance the endurance and efficacy of restorations by necessitating less restorative material, hence promoting the overall oral and systemic health of young patients.

The limitation of the present study is that the results derived are commonly attributed to the *in vitro* setup used in the present study. However clinical trials and *in vivo* assessment of the quality of obturation could provide us with an insight over the clinically relevant aspects. Apart from preparation and obturation volume analysis, canal centering ability, transportation, uninstrumented regions, microcracks can also be assessed for obtaining further knowledge in the mechanical aspects. Use of different tapers of the file systems used could provide an overt expression of the results towards the more tapered file system. Also, the curvature of the roots was not standardized before the start of the study. This could also have had an influence as the more curved canals could have had a minimal preparation and obturation volume due to unavoidable human errors. Hence further studies are recommended keeping the above limitations to obtain a proper method for measuring the volumetric changes.

## 5 Conclusion

The present study suggests minimal preparation volume and obturation volume using rotary file systems compared to hand-K file system. Kedo-S square showed the least difference in preparation volume and better obturating volume compared to Pro AF Baby Gold file systems.

## Data Availability

The original contributions presented in the study are included in the article/Supplementary Material, further inquiries can be directed to the corresponding authors.
